# A nationwide survey of Antimicrobial Stewardship in Pediatric Intensive Care Unit: implementation notes from the Brazilian underground

**DOI:** 10.1017/ash.2023.530

**Published:** 2024-01-03

**Authors:** Eliane Carlosso Krummenauer, Henrique Ziembowicz, Mara Rubia Santos Gonçalves, Magda Machado de Miranda Costa, Mariana Portela de Assis, Viviane Maria de Carvalho Hessel Dias, Cláudia Fernanda de Lacerda Vidal, Rochele Mosmann Menezes, Jane Dagmar Pollo Renner, Marcelo Carneiro

**Affiliations:** 1 Santa Cruz Hospital and Postgraduate Program in Health Promotion, University of Santa Cruz do Sul, Rio Grande do Sul, Brazil; 2 Brazilian Association of Professionals in Infection Control and Hospital Epidemiology, Stewardship Brazil Group, Brazil; 3 University of Santa Cruz, Rio Grande do Sul, Brazil; 4 National Health Surveillance Agency, Ministry of Health, Distrito Federal, Brazil; 5 Nossa Senhora das Graças Hospital, Paraná, Brazil; 6 Federal University of Pernambuco, Pernambuco, Brazil; 7 Postgraduate Program in Health Promotion, University of Santa Cruz do Sul, Rio Grande do Sul, Brazil

## Abstract

A multicenter diagnostic study was conducted to investigate the implementation of an Antimicrobial Stewardship Program in Brazilian Pediatric Intensive Care Units. The analysis unveiled the main implementation impediments of the Antimicrobial Stewardship Program such as the lack of professionals and resources available to the program.

## Background

Antimicrobial Stewardship Programs (ASPs) aim to reduce costs, optimize therapeutic outcomes, and mitigate Antimicrobial Resistance (AMR).^1,2^ The emergence and dissemination of AMR are primarily influenced by medical prescription habits and breaches of infection prevention and control. In response, ASPs have been established in healthcare institutions to optimize Antimicrobial (ATM) prescribing behaviors and dispensing practices and ensure favorable clinical, pharmacoeconomic, and management outcomes.^1,2^

The establishment, regulation, and inspection by local regulatory authorities of hospitals’ compliance with the ASPs are based on a recent norm, which is why this study is the first in PICUs to evaluate the implementation of actions in Brazil (BR).^3^

All of these points are strained and grounded in the vast Brazilian context, especially after the pandemic, a country of continental size. To emphasize, in the year 2022, BR had 4.466 active hospitals with 263.793 functioning beds in operation only in the private healthcare network, numbers that exceeded the pre-pandemic period.^4^

The BR National Health Surveillance Agency (ANVISA) has reported that there are 2.175 hospitals in BR’s Intensive Care Unit beds (ICU). Of these, 1.982 were adult patients, 593 were pediatric patients, and 766 were neopediatric units, totaling 3.341 ICU beds. Brazil has demonstrated a trend in the number of doctors and the ratio of doctors to 1.000 inhabitants, with an increase from 1.6 in 2010 to 2.6 in 2023.^5^ Nevertheless, significant regional disparities persist.

With this in mind, this nationwide survey aims to describe the barriers to implementing ASPs in Pediatric Intensive Care Units (PICUs) in BR hospitals.

## Methods

This cross-sectional survey was conducted between October 2022 and January 2023. The participating hospitals were recruited through an official invitation from ANVISA. The study was voluntary, utilizing a validated instrument to analyze the implementation of ASPs in PICUs in BR hospitals. The instrument’s consistency analysis had already been carried out in a previous survey of ASP in adult ICUs using Cronbach’s alpha, with satisfactory results, as four components were considered good (α > 0.8) and one was excellent (α > 0.9).^6^ In this study, the collected characteristics included the reasons that contributed to the hospital’s non-implementation of ASPs and the actions taken to monitor the use of antimicrobials in the PICUs. The data collected through a multiple-choice questionnaire, analyzed using Statistical Package for the Social Sciences version 23, allowed participants to select multiple answers over time.

## Results

**Figure 1.**
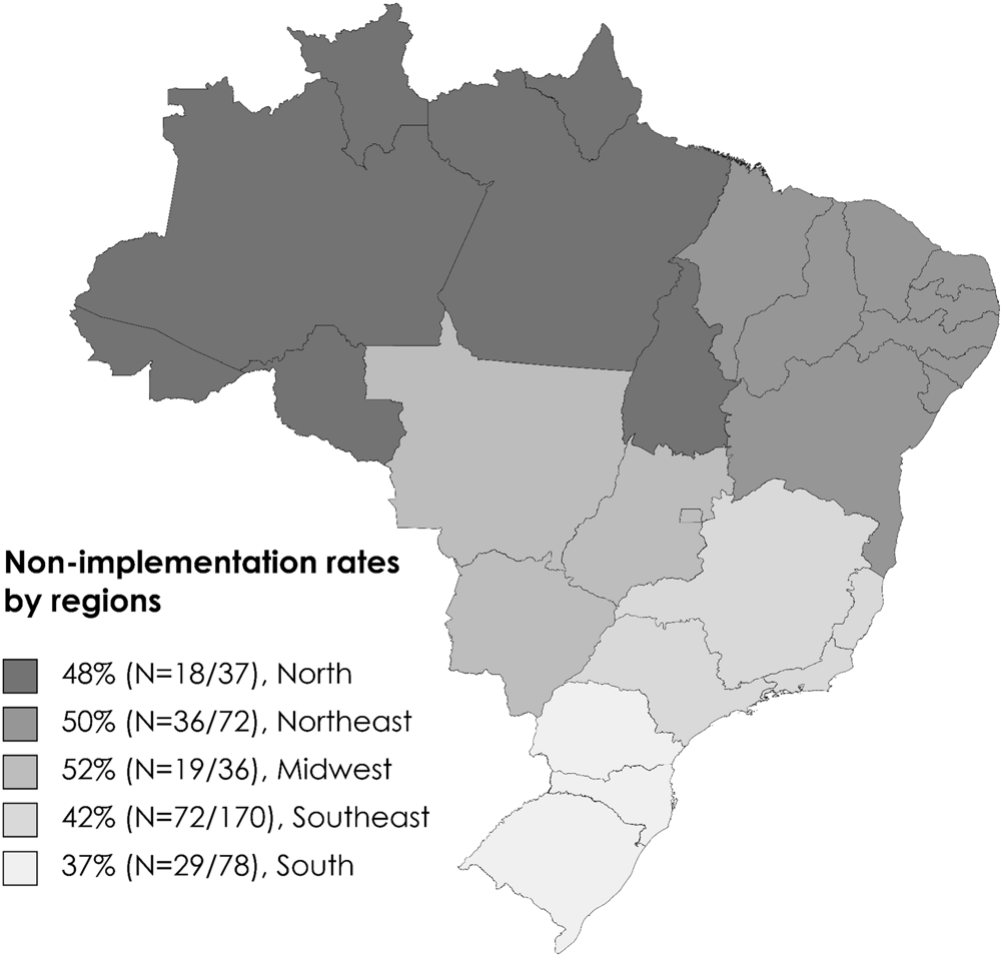
The non-implementation rates of Antimicrobial Stewardship Program in hospitals with Pediatric Intensive Care Units in Brazil by political-administrative regions.

Of the 593 hospitals with PICUs in BR, a response rate of 66.3% (N = 393) was obtained for the survey. It was found that 44.3% (N = 174) of the hospitals had not yet implemented an ASP (Figure 1).

It was observed that 41.4% (N = 72) of the hospitals without an ASP were affiliated with the Brazilian public healthcare system, while 58.6% (N = 102) were affiliated with the complementary healthcare system, which includes private facilities. The analysis of barriers to ASP implementation, with 317 responses, was based on a multiple-choice question regarding the reasons contributing to the hospital not having developed or implemented an ASP.

Regarding the responses (Table 1), we noticed the following reasons: insufficient number of professionals for ASP implementation for 53.5% (*N* = 103); lack of support from hospital departments involved in the process, such as pharmacy and laboratory for 27.8% (*N* = 49); lack or scarcity of technological resources, 29.9% (*N* = 58); lack or scarcity of financial resources to 28.5% (*N* = 54); absence of trained healthcare professionals for ASP implementation to 18.7% (*N* = 29); absence of information technology support to 7.4% (*N* = 20); insufficient support from hospital management to 9.6% (*N* = 19); and ASP still in the implementation phase to 4.4% (*N* = 14). The remaining responses, 1.6% (*N* = 5), were destined for the “others” category.

**Table 1.** The non-implementation rates of Antimicrobial Stewardship Programs in hospitals with Pediatric Intensive Care Units in Brazil by political-administrative regions

Item evaluated	Brazil by political-administrative regions				
	North	Northeast	Midwest	Southeast	South
	*N* = 18 (%)	*N* = 36 (%)	*N* = 19 (%)	*N* = 72 (%)	*N* = 29 (%)
Absence of trained healthcare professionals for ASPs implementation	8 (44.4)	10 (27.8)	0 (0)	8 (11)	3 (10.3)
Insufficient number of professionals for ASP implementation	8 (44.4)	22 (61)	5 (26.3)	48 (66.7)	20 (69)
Lack of information technology resources	1 (5.5)	4 (11)	0 (0)	15 (21)	0 (0)
Lack of support from hospital departments involved in the process, such as pharmacies and laboratories	6 (33.3)	16 (44.4)	4 (21)	19 (26.4)	4 (14)
Lack or scarcity of financial resources	6 (33.3)	12 (33.3)	4 (21)	27 (37.5)	5 (17.2)
Insufficient support from hospital management	1 (5.5)	4 (11)	1 (5.3)	9 (12.5)	4 (14)
Lack or scarcity of technological resources	4 (22)	16 (44.4)	4 (21)	27 (37.5)	7 (24)

## Discussion

A national evaluation of ASP implementation in PICUs in BR revealed limited implementation. Since the Infectious Diseases Society of America guidelines for ASP development were published in 2007, there has been an increase in the number of formal ASPs in pediatric hospitals. This was partly due to the increased recognition of unique childhood factors. Pediatric ASP specialists emphasize that children are different from adults, that ASP members must be well-informed about infectious diseases and medication properties, and that certain adverse effects are more common in children.^7^ Our findings align with those stated in a systematic review by Nathwani et al. (2019), who suggested that the successful implementation and sustainability of an ASP may require the allocation of additional resources, such as hospital staff and equipment.^8^

Brazil’s National Health System aims for universal coverage but faces unequal access due to socioeconomic and structural inequalities. The southeast region has more health infrastructure, whereas the southern region has better technology and more medical centers, leading to better health indicators. The southeastern and southern regions have better health indicators; however, indigenous people also face unique challenges.

Urban areas in the Midwest have better healthcare availability than rural areas that lack resources. The Northeast region faces difficulties in accessing healthcare, which results in a shortage of specialized medical care and elevated infant mortality rates. Brazil has experienced disparities in healthcare, with some states having fewer doctors per patient due to variations in economic development and policies.

Access to healthcare in the northern regions is limited. The North and Northeast regions have fewer doctors and lower ratios than the Southeast region, which has the highest ratio.

In 2017, ANVISA published the “National Guideline for Antimicrobial Use Management in Health Services” to encourage BR health services to design and implement effective programs. The guideline outlined the essential components of an effective program. In the new guideline 2023 version, however, the gaps, potential solutions, and strategies to overcome them are not well elucidated in a practical way.^9^

Our study identified ASP structure and resources as the main barriers to implementation. Indeed, this was not surprising, especially in the Brazilian LATAM context, which is stuffed with inequalities. The 2023 guidelines provide recommendations for implementing effective programs and offer strategies for overcoming potential barriers.^9^ Senior hospital management must support ASPs by allocating resources and encouraging engagement, especially with prescribers. Success relies on participation from senior management, clinical, nursing, and pharmacy teams. Incorporating ASPs into strategic objectives shows strong commitment, and leaders should increase awareness and include ASPs in their routines. The management team’s expertise, care team involvement, and hospital culture play a role in several key processes, including prospective audits and feedback, adjustment of drug dose or duration, conversion to oral formulations, assessment of drug interactions, review of positive cultures with de-escalation opportunities, rapid molecular tests, and ATM management at discharge.

On the other hand, scientific literature indicates that the presence of ASP in PICUs is limited, and only a few programs adhere to all of the existing recommendations.^10^

Indeed, the recognition of the need for a formal ASP in pediatrics has only recently been acknowledged, taking into account the extensive utilization of antibiotics in children and the distinct AMR patterns observed in this population compared to adults and the elderly.^7–10^ Herein, on the Brazilian underground, the authors of this survey tend to indicate that insufficient support from hospital management and lack of support from hospital departments involved in the process must not undermine the ASP implementation. Conversely, the team’s willpower conveys the ability to suppress threats, while simultaneously seizing opportunities at the same pace.^9^ Albeit, we must keep it real: the lack or scarcity of technological resources and financial resources are the quicksand of the Brazilian context.
